# Antecedents and Consequences of Information Overload in the COVID-19 Pandemic

**DOI:** 10.3390/ijerph17249305

**Published:** 2020-12-12

**Authors:** Hyehyun Hong, Hyo Jung Kim

**Affiliations:** 1 Department of Advertising & Public Relations, Chung-Ang University, Seoul 06974, Korea; hhong@cau.ac.kr; 2 Department of Media & Communication, Pusan National University, Pusan 46241, Korea

**Keywords:** information overload, information processing, cognitive capacity, behavioral intention, COVID-19

## Abstract

The global outbreak of coronavirus disease (COVID-19) in 2020 has significantly affected the information environment as well as the daily life of individuals across the world, with information about COVID-19 dominating all media channels. The information provided at the time of a health crisis like COVID-19 is critical in helping people learn about the disease and the recommendations to prevent infection. However, studies have shown that when people are overwhelmed by too much information (referred to as ‘information overload’), this leads to adverse effects. This study examined the antecedents and consequences of information overload in the context of COVID-19. A survey was conducted among 627 residents in Seoul, South Korea, one of the earliest affected countries in the global outbreak. The results showed that cognitive capacity and the frequency of online news use and interpersonal communication were significant predictors of information overload. Information overload influenced how information is processed; it was associated with the tendency toward greater heuristic processing and less systematic processing. In addition, people were more likely to enact prevention behaviors when the information was processed systematically, as opposed to heuristically. The results are discussed considering both the theoretical and practical implications.

## 1. Introduction

In the past few decades, no issue has received greater global attention than coronavirus (COVID-19 hereafter). Since its first outbreak in December 2019, COVID-19 has affected and is still affecting significant numbers of people across the world. As of October 2020, over 37 million cases were confirmed with about one million deaths globally according to the World Health Organization (WHO) [1]. The severity of COVID-19 on a global scale and its significant impacts on individual lives have generated intensive media coverage. New information has been presented every minute to deliver the fast-changing statistics on COVID-19, daily briefings by governments, and experts’ commentaries. It is not an overstatement that information about COVID-19 has dominated all online and offline media since WHO officially announced it as a global pandemic in March 2020.

Providing health information is crucial because it helps people attain proper knowledge and make informed decisions about their health. Particularly, in times of health crises such as COVID-19, people become very attentive to such information and, by doing so, try to reduce uncertainty and negative feelings associated with the previously unknown disease. However, studies have shown that when individuals are overloaded beyond capacity, adverse effects such as information avoidance, confusion in decision making, and lack of compliance with recommended behaviors may emerge [2,3,4,5]. Moreover, individuals of older age and lower education level and those with less knowledge or poorer cognitive abilities are more vulnerable to information overload and its negative effects [6,7,8,9]. Despite the potentially negative impacts of information overload, the scope of previous studies on this issue is limited. Specifically, in a recent systematic review, Khaleel et al. [10] pointed out that most research on health information overload has been conducted in the U.S. with a focus on the ‘cancer’ information overload perceived by cancer patients, and thus called for extending the scope to other health issues in different contexts.

The abundance of information about the on-going COVID-19 pandemic across a wide range of communication channels—from traditional media to online platforms—provides a sufficient rationale to examine the impacts of information overload in this challenging time. This study was conducted in the context of COVID-19 in South Korea, which was one of the earliest affected countries in the global outbreak. South Korea is also well-equipped with mobile technology and network infrastructure, which enabled a huge amount of information to be quickly delivered and shared with people. In particular, the use of social media has become popular in South Korea, with a penetration rate of 87%, following that of the United Arab Emirates (99%) and Taiwan (88%). The penetration rate is about 1.8 times higher than the global average (49%) [11]. Nearly 90% of Korean people aged over 13 years old report using at least one social media channel from the wide variety of mobile messaging, social networking, and video-sharing platforms available [12]. In the information environment saturated by COVID-19, the current study aimed to identify the antecedents and consequences of information overload at the individual level.

## 2. Literature Review

### 2.1. Health Information Overload and COVID-19

With the development of online communication channels and the substantial amount of health information available as a result, the phenomenon of information overload has received significant attention from researchers and practitioners in the field of public health and health communication [10,13]. Specifically, information overload refers to the state of feeling overwhelmed by the amount of information presented, such that it is too much process [4,14]. Information overload is cultivated by exposure to information from diverse mediated channels and personal sources (e.g., healthcare providers, everyday conversations with other people) [4], and it is aggravated when information arrives from multiple formats and channels [15].

However, scholars also defined information overload in relation to the quality of information, the cognitive or emotional responses toward information, and its outcomes [6,9,16,17]. For example, whereas Ji et al. [18] emphasize that too much information cannot be processed in a limited time frame, Eppler and Mengis [16] emphasize the role of the quality of information, which is often associated with uncertainty, ambiguity, novelty, and complexity of information. Kim and colleagues highlight the adverse outcomes of information overload on decision making, by defining it as “a perception of being overwhelmed and confused by information coming in that might hinder learning or impair users’ ability to make informed decisions” ([9], p. 4). While most definitions deal with cognitive aspects (e.g., the amount of information beyond one’s ability to process), Schommer et al. [17] focused on emotional responses, such as confusion, frustration, doubtfulness, anger, and vulnerability, toward the inundated information. Additionally, in the cancer context, Chae et al. defined information overload as “an aversive disposition wherein a person is being confused and overwhelmed by cancer information, which occurs when he or she fails to effectively categorize new information due to a lack of resources for effective learning” ([6], p. 626). By reviewing previous definitions, Kim et al. [9] summarized the key components of information overload as follows: (a) overflow of information, (b) ineffective management of information due to limited capacity, (c) stress or anxiety, and (d) ambiguity. In the current study, we also adopted this comprehensive definition of information overload.

The model of information overload [19,20] explains the nature of information overload by relying on the limited cognitive capacity of human beings, which hinders the proper processing of information-namely, encoding, storage, and retrieval. According to Lang [21], cancer information needs large amounts of cognitive resources for individuals to understand and store the information in memory because of its highly arousing and complex content. We speculate that COVID-19 information is similar in such characteristics, and thus those who process information about COVID-19 demand greater resources to process the information and are vulnerable to information overload. In the same vein, Eppler and Mengis [16] argued that specific characteristics of information are related with information overload. Their contention is well-suited to information about COVID-19 in that it is characterized by a high level of uncertainty (COVID-19 was an unknown and unexplored disease before its outbreak), ambiguity (governmental guidance for prevention has shifted over time and experts and authorities have presented different perspectives, particularly in the early stage of the outbreak), and complexity (information about COVID-19 contains a lot of scientific jargon), all of which can contribute to increasing the occurrence of information overload.

### 2.2. Antecedents of Information Overload

According to Kim et al.’s [9] conceptual framework, information overload is based on the exposure to information and experience of information seeking, and thus is often influenced by individual properties, such as socio-demographics and health status. As for the influence of socio-demographics, studies have shown relatively consistent results. Generally, those who are older [7,20], less educated [6,7,9,22], and of lower socioeconomic status [8,9] perceive greater information overload. People in poorer physical and mental health conditions also tend more to suffer from information overload [7,9], and trait anxiety also successfully predicts information overload [2,6].

In addition, people who have lower health literacy or confidence in health information seeking express greater information overload [7,8,9,18]. Information overload is negatively related to the level of relevant knowledge about an issue, such as that of sun-safe protection [14] and anticoagulants [22]. These studies support that an individual’s capacity for information gathering, processing, and understanding is highly associated with the occurrence of information overload. Cognitive capacity is directly related to an individual’s ability and motivation to handle information properly and effectively. Therefore, people with a lower level of capacity have a greater tendency toward being overloaded by information because new information is poorly understood or incorporated into existing knowledge [6,19]. Kim et al. [9] stated that an examination of information ability is important because a lack of ability in this regard is highly relevant to those who are at the greatest health risks in society. Although different terms have been used to indicate an individual’s information-related ability (e.g., health information literacy, search expertise, self-efficacy, confidence in finding information, information searching efficiency, perceived information-gathering capacity), its influence on information overload has been reported consistently in previous studies. We employ the term ‘information ability’ to refer to an individual’s ability for information search and understanding.

Although information overload is often considered to be a by-product of the information-seeking process in a saturated media environment [9,20], not many studies have examined the influence of media use on information overload and additionally, the results have been inconsistent across studies. For example, regarding information about diet and nutrition, Ramirez and Arellano Carmona [23] showed that the perception of information overload was prevalent among Mexican-American females and this was mostly attributed to media presentations, in which the effects of too much and contradictory information are compounded. Kim et al. [9] considered media use (i.e., television, radio, newspapers, magazines and the Internet) as a potential predictor of information overload, but found no evidence that greater attention to media caused greater information overload. In predicting perceived information overload, Chae et al. [6] included the use of nine media channels for health information across four categories: (a) print (newspapers and magazines or newsletters), (b) television (news and health programs), (c) the Internet (online newspapers, professional health-related websites, social networking sites or online communities), and (d) interpersonal communication (family/friends and health care professionals). Among the four, the use of print media and the Internet were both negatively associated with cancer information overload in three out of the four samples under study. Chae et al. [6] explained that, compared to television, print media and the Internet are active channels that require greater cognitive effort, and are often used by highly motivated and engaged individuals. Ji et al. [18] also examined the relationship between news media use and information overload, although not in the health context. They found a partially inverted U-shaped relationship regarding traditional news media use and information overload, but the use of Internet news media was not related to information overload.

Based on the aforementioned literature, a research question was posited to explore the potential predictors of information overload in the context of COVID-19 as follows:

**RQ:** Does information overload differ across individual socio-demographic characteristics (i.e., age, gender, education level, and income level), cognitive capacity (i.e., current knowledge and information ability), and media use when obtaining COVID-19 information?

### 2.3. Consequences of Information Overload

#### 2.3.1. Influence on Information Processing

Previous studies have shown that information overload affects individuals across diverse dimensions. First, it is highly related to an individual’s emotional state. According to Swar, Hameed, and Reychav [24], information overload has an impact on psychological health, including negative affect, depressive symptoms, anxiety, and anger, all of which mediate its negative impacts on an individual’s intention to search online health information. As such, studies have provided evidence on the impact of information overload on information search behavior, particularly regarding information avoidance [2,3,14,25]. According to Chae [2], information avoidance is not just the opposite of information seeking but is a deliberate response to the information provided to reduce (a) uncertainty and distress, (b) conflicts with previous knowledge, and (c) cognitive efforts to understand and interpret. Thus, the relationships between information overload and avoidance can be explained from both cognitive and affective perspectives. Briefly, people avoid information to reduce cognitive burden and/or to minimize uncomfortable emotional states, such as stress and confusion that accompany information overload [2,3].

The underlying mechanism between information overload and avoidance can be partly explained by the heuristic-systematic model (HSM), which is a dual-processing framework within limited cognitive resources. According to Chaiken and colleagues [26,27,28], HSM was originally developed to explain attitude changes based on persuasive messages as a result of two modes of information processing: heuristic vs. systematic processing. Systematic processing involves “attempts to thoroughly understand any and all available information through careful attention, deep thinking, and intensive reasoning” whereas heuristic processing relies on heuristic cues (e.g., characteristics of source or arguments) or simple judgmental rules (e.g., “the expert knows best”) [26] (p. 247). Because systematic processing demands a substantial amount of effort, individuals should be capable and motivated in devoting their attention to information. On the other hand, heuristic processing requires less or minimal effort and thus less dependent on one’s ability and motivation, and it sometimes occurs automatically [26,28].

Eppler and Mengis [16] explained how an individual experiences processing information when his/her capacity is exceeded by the information flow as follows: “the individual confronts problems in identifying relevant information, becomes overtly selective and neglects a large amount of information, faces difficulties in understanding the association between details and the overall perspective” (p. 417). All of the noted symptoms are related to the heuristic processing of information. As information overload is the state of lacking cognitive resources to process information, it is expected that people who experience information overload will engage more in heuristic processing and less in systematic processing. Therefore, the first hypothesis posited:

**H1a.**  *Information overload will be positively associated with heuristic information processing.*

**H1b.**  *Information overload will be negatively associated with systematic information processing.*

#### 2.3.2. Influence on Behavioral Intentions

Studies have shown that information overload is negatively related to the performance of health behaviors and behavioral intentions. For example, those who perceive higher information overload are less likely to perform regular medical checkups and cancer screening, such as colonoscopy and mammography [25,29] and less willing to engage in cancer prevention behaviors (e.g., sunscreen use, not tanning) [5,14]. In addition, information overload negatively influences healthy lifestyle behaviors, such as the consumption of fruits and vegetables and regular exercise [5]. Although little has been documented on behavioral intentions to prevent COVID-19, Jimenez et al. [30] recently showed that a belief associating coronavirus with death negatively influences an individual’s intention to take preventive behaviors, such as social distancing and handwashing. Thus, if the overloaded information generates such a belief, it also has a potency to reverse the willingness to perform prevention behaviors. Thus, based on the literature aforementioned, a negative relationship between information overload and behavioral intention was hypothesized as follows.

**H2.**  *Information overload will lead to lower intentions to perform prevention behaviors.*

**Figure 1:**
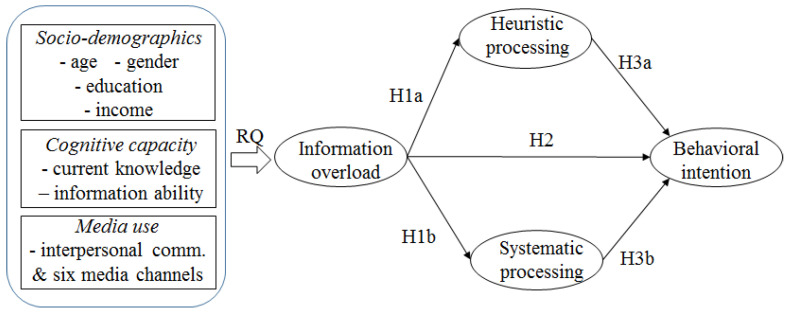
Research model.

While previous studies have consistently reported a negative relationship between information overload and behavioral intention, information processing may be an underlying mechanism through which information overload influences behavioral intention. Going back to HSM, one of the major speculations is that the information processed (particularly through a systematic mode) is used to formulate an individual’s subsequent attitudes, judgments, and behaviors [26]. Within the HSM framework, the antecedents and consequences of information processing have been tested in the context of health risks (e.g., [31,32]). Ryu and Kim [31] showed that, regarding information about nuclear power accidents, systematic processing was influenced significantly by receiver’s characteristics, such as motivation, involvement, and ability, rather than other message- or source-related factors, while heuristic processing was largely affected by the vividness of information. In processing information, males were more likely to engage in a systematic mode and those who were older and with less income in a heuristic mode. Furthermore, systematic processing significantly led to the perception of greater risk associated with a nuclear power accident. Similarly, Trumbo [32] showed that heuristic processing of information about cancer rates decreased risk perception while systematic processing increased risk perception across three samples in the study. Although these studies do not provide a clear link between information processing and behavioral intentions, it is logical to predict that risk perception aggravated by systematic processing would lead to a greater intention to perform prevention behaviors. Corresponding to the HSM speculation that systematic processing, as opposed to heuristic processing, contributes more to behavioral changes as intended by the message [26], the last hypotheses were proposed relating to the impact of information processing on behavioral intentions, and the overall research model is provided in Figure 1.

**H3a.**  *Heuristic processing will lead to lower intentions to perform prevention behaviors.*

**H3b.**  *Systematic processing will lead to higher intentions to perform prevention behaviors.*

## 3. Materials and Methods

### 3.1. Survey Procedure and Sample

An online survey was conducted in South Korea from 20–24 March 2020. Since the first outbreak on 20 January 2020 (via a Chinese woman who entered South Korea on an airplane), most of the people with COVID-19 were either from Wuhan, China, or those who had been in contact with a person with COVID-19 in January and February 2020. As more than 100 individuals per day were repeatedly confirmed, the Korean government announced the highest level of infectious disease warning (red level) on 23 February 2020 and mandated official shutdown of public spaces, such as public schools, daycare centers, and sports facilities. According to the Korean government, the cumulative number of people with COVID-19 reached 8652 (of which 94 were dead) by 20 March 2020.

Upon the researchers’ request, a research firm developed an online survey site and sent an e-mail invitation to its 3000 panelists who were residing in Seoul, South Korea. The online panel used in the current study is the biggest panel in South Korea, with around 1.3 million panelists as of February 2020 [33]. The panelists are recruited via a variety of online and offline methods, including random-digit-dialing, voluntary registration, and the recommendation of other panelists. The identity and demographics of panelists were verified and a training session required at the registration stage. To ensure sample quality, panelists are rewarded based on the firm’s loyalty programs and their personal information is updated on a regular basis.

The e-mail invitation in this study provided brief information about the research topic, the length of the survey, and the level of monetary reward. When the panelists visited the online survey site, a more detailed description about the study (e.g., the purpose of the study) and a consent form were provided along with statements assuring the principles of voluntary participation, anonymity, and confidentiality. Only those who agreed were directed to the main questionnaire, and a total of 627 respondents completed the survey. By employing a quota sampling technique based on gender and age, the number of males (*n* = 312, 49.8%) and females (*n* = 315, 50.2%) was balanced, and age (*M* = 40.34, *SD* = 11.866; *range* = 20–79) was distributed equally across those aged in their 20s (*n* = 153, 24.4%), 30s (*n* = 155, 24.7%), 40s (*n* = 159, 25.4%), and 50 or over (*n* = 160, 25.5%). For the level of education, the vast majority were college graduates or current attendees (*n* = 443, 70.7%), followed by post-graduates (*n* = 101, 16.1%) and high school graduates or less (*n* = 83, 13.2%).

### 3.2. Measurements

#### 3.2.1. Perceived Information Overload

Seven items were used to measure perceived information overload on a five-point Likert scale. The items were modified from previous studies (mainly, Jensen et al.’s cancer information overload scale [4]) to fit with the COVID-19 context. They included: “I had no idea how to check all the information about COVID-19 due to abundance of information,” “There is so much information about COVID-19, it’s hard to know which to follow,” “Information about COVID-19 all starts to sound the same after a while,” “There is so much information about COVID-19, I forget most information about COVID-19 right after I hear it,” “I feel overloaded by the amount of COVID-19 information I am supposed to know,” “It is hard to know which information about COVID-19 is accurate and trustworthy,” and “I feel confused by a lot of conflicting information about COVID-19” (*M* = 3.14; *SD* = 0.715; *α* = 0.862).

#### 3.2.2. Information Processing

How information is processed (systematically vs. heuristically) was measured on a five-point scale using six items, adapted from Yang et al. [34]. For systematic processing, three items, including “I try to think thoroughly to better understand the information about COVID-19,” “I found myself paying attention to the information about COVID-19,” and “I try to think about how the information about COVID-19 related to other things I already know” were used (*M* = 3.67; *SD* = 0.676; *α* = 0.794). Heuristic processing was measured by asking how much respondents agreed with the three statements: “I do not spend much time thinking about the COVID-19 information,” “When I read or listen to the information about COVID-19, I skim through the information,” and “When I encounter a news story about COVID-19, I focus on only a few points” (*M* = 2.64; *SD* = 0.834; *α* = 0.820).

#### 3.2.3. Behavioral Intentions

Behavioral intentions measured the degree to which individuals intended to follow the recommended behaviors to prevent COVID-19 on a five-point scale (1 = not at all to 5 = very much). The items were developed based on the official guidelines from the Korean government and WHO for COVID-19 prevention. Specific items measured their willingness to avoid public spaces where many people visit; to wear a mask when being out of home; to wash hands frequently; and to keep social distancing (*M* = 4.44; *SD* = 0.612; *α* = 0.859).

#### 3.2.4. Cognitive Capacity

Individual cognitive capacity was measured in two dimensions: (a) the estimation of current knowledge, and (b) an ability to search and understand information (i.e., information ability). For current knowledge, adopting the approach of Yang and colleagues [34,35], respondents were asked to estimate their knowledge of COVID-19 between 0 (knowing nothing) and 100 (knowing everything). An average of current knowledge was 73.22 (*SD* = 15.68). Information ability was measured by four items, which were modified from Yang et al.’s [34] measures for information-gathering capacity. Respondents rated on a five-point scale (1 = not at all to 5 = very much) how much they agreed with the following statements: “It is difficult to obtain useful information about COVID-19 (reverse coded),” “Most information about COVID-19 is too technical for me to understand (reverse coded),” “When it comes to information about COVID-19, I don’t know how to separate facts from fiction (reverse coded),” and “I know what to do when I need further information about COVID-19” (*M* = 3.32; *SD* = 0.709; *α* = 0.732). Responses were averaged and greater scores indicated higher ability regarding information search and comprehension.

#### 3.2.5. Media Use

Media use was measured on a five-point scale (1 = not at all to 5 = very often) by asking how frequently respondents used each of seven sources of information regarding COVID-19: (a) interpersonal (family, friends, co-workers, and other acquaintance) (*M* = 3.38; *SD* = 0.893), (b) television news (*M* = 4.11; *SD* = 0.937), (c) newspaper (*M* = 2.22; *SD* = 1.255), (d) online news (*M* = 4.19; *SD* = 0.793), (e) social media (*M* = 3.19; *SD* = 1.108), (f) mobile applications designated for COVID-19 information (e.g., Corona Doctor, Corona Map, Corona Now, etc.) (*M* = 2.80; *SD* = 1.241), and (g) government channels (e.g., official websites, SMS) (*M* = 3.32; *SD* = 1.151).

#### 3.2.6. Socio-Demographics

Respondents identified their socio-demographic status, including age, gender (1 = male, 2 = female), education (1 = high school graduate or less, 2 = college graduate or currently attending, 3 = postgraduate), and household income in KW (1 = Less than 1000 k, 2 = 1000 k to under 2000 k, 3 = 2000 k to under 3000 k, 4 = 3000 k to under 4000 k, 5 = 4000 k to under 5000 k, 6 = 5000 k to under 6000 k, 7 = 6000 k to under 7000 k, 8 = 7000 k to under 8000 k, 9 = 8000 k or more).

## 4. Results

To answer the research question, a hierarchical regression analysis was performed as shown in Table 1. The first model included four socio-demographic variables (i.e., age, gender, educational level, and income level) but did not explain much of the variance in information overload (*F* = 0.301, *p* = 0.878; *R*^2^ = 0.002). None of the four variables was a significant predictor. However, when the independent variables of cognitive capacity and media use were added, the model became significant (*F* = 21.371, *p* < 001), and its explanatory power increased to 31.2%.

Specifically, an individual’s information ability was the strongest predictor (*β* = −0.519, *t* = −13.753, *p* < 0.001), indicating that those who have greater ability to search and process information were less likely to perceive information overload, which supports the limited capacity of a human being as presented by the information overload model. The amount of current knowledge was also inversely related to information overload (*β* = −0.073, *t* = −1.976, *p* < 0.05), and the more knowledge an individual estimated they had, the less likely information overload was perceived. Among the media use variables, two channels—interpersonal communication and online news—were associated with information overload. Higher scores in information overload were reported as individuals used more interpersonal communication (*β* = 0.115, *t* = 3.268, *p* < 0.01) and online news (*β* = 0.100, *t* = 2.725, *p* < 0.01). The use of other media channels (i.e., TV news, newspaper, social media, mobile apps, government channels) did not predict information overload.

To examine the influence of information overload on information processing and behavioral intention, a structural equation model (SEM) was tested using the AMOS program. Overall goodness-of-fit indices showed that the proposed model was well-fitted to the current data (χ^2^_(112)_ = 422.219, *p* < 0.001; χ^2^/*df* = 3.770; GFI = 0.929, AGFI = 0.903; NFI = 0.918; CFI = 0.938; RMSEA = 0.076; RMR = 0.067). In addition to the hypothesized paths, two arrows for covariance were added based on the results of the model modification indices. One was between the error terms associated with systematic processing and heuristic processing, and the other one was the link between the two behavioral intention measures (BI1 and BI4).

**Figure 2:**
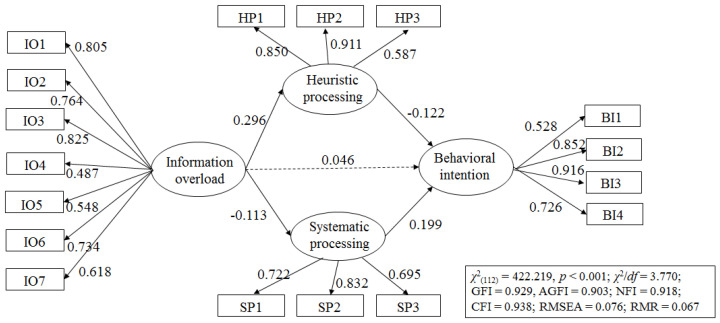
Results of structural equation modeling. Note. IO = information overload; HP = heuristic processing; SP = systematic processing; BI = behavioral intention. The solid lines represent significant relationships, while a dashed line represents a non-significant relationship between variables. Rectangles indicate measured items for variables shown as ovals.

As seen in Table 2 and Figure 2, the influences of information overload on the two modes of information processing were significant, supporting the first set of hypotheses (H1a and H1b). Specifically, information overload was positively associated with heuristic processing (*β* = 0.296, *p* < 0.001; *B* = 0.416, *SE* = 0.066) whereas it was negatively with systematic processing (*β* = −0.113, *p* < 0.05; *B* = −117, *SE* = 0.049). The relationship between information overload and behavioral intention was not significant (*β* = 0.063, *p* > 0.05; *B* = 0.046, *SE* = 0.032), and thus, H2 was not supported. However, the way information was processed significantly influenced behavioral intention as hypothesized (H3a and H3b). Heuristic information processing weakened an individual’s intentions to engage in prevention behaviors (*β* = −0.237, *p* < 0.001; *B* = 0.122, *SE* = 0.029) while systematic information processing strengthened behavioral intention (*β* = 0.285, *p* < 0.001; *B* = 0.199, *SE* = 0.041).

## 5. Discussion

The COVID-19 pandemic may be the most critical health issue of the 21st century. All public and media attention has been directed toward this previously unknown disease, and the public has received a tremendous amount of related information. New information has been released throughout and numerous media channels have been saturated with COVID-19 information from diverse sources. Accurate and reliable information helps the public make informed decisions about their health, including those relating to preventive behaviors, early detection, and choice of treatment options [36]. Nevertheless, there have been no systematic studies yet on what factors affect information overload and what consequences it potentially has in the context of COVID-19. The current study aimed to fill this void by testing the influence of potential predictors of information overload and the impacts on information processing and behavioral intentions. Key results are discussed and elaborated from both theoretical and practical perspectives in the following sections.

### 5.1. Antecedents of Information Overload

Contrary to previous studies, there were no socio-demographic differences in information overload found in the current study. Presumably, this results from the timing of the study because the survey was conducted at the peak of media coverage and public attention to COVID-19, and at the start of strong governmental regulation for disease prevention (e.g., the shutdown of public spaces). Given the dominance of COVID-19 issues in Korean society, it seems possible that there was a ceiling effect that most people, if not all, were attentive to the COVID-19 issue and sought information about it.

However, corresponding to previous studies [7,8,9,14,18,22], variables regarding cognitive capacity were significant predictors of information overload. While both information ability and current knowledge were negatively associated with information overload, the effect size for information ability was far greater than that for current knowledge. The results support the prediction of the information overload model that information overload occurs when the flow of incoming information exceeds an individual’s capacity for information management. The negative directional influence confirms that the threshold of occurring overload was higher among those with greater cognitive capacity.

Among many communication channels, only two measures—the frequency of online news use and interpersonal communication—were significantly related to information overload. In previous studies, the relationship between media use and information overload was not consistent. A few studies addressed an insignificant influence of online platforms (e.g., the Internet) (e.g., [9,18]), but others found a negative influence (e.g., [6]). The current study tried to delve into this by separating the influences of specific channels. As a result, those who consumed online news frequently were more likely to perceive information overload although most of the other online channels, such as social media and mobile applications, were not significantly associated with information overload. It is worth noting that respondents used online news channels most frequently (*M* = 4.20, *SD* = 0.793), in which information about COVID-19 was being updated continuously with intensive repetition by multiple online news outlets. The results of the current study imply that online news channels contribute considerably to the environment of information overflow while leading to information overload.

In addition, those who engaged more in interpersonal communication about COVID-19 also tended to perceive greater information overload. One possible reason is that personal communications with others have increased the amount of information that requires cognitive effort to process. In the same vein, Kim et al. [9] showed a significant relationship between overload and reliance on proxy personal sources (e.g., family and friends) for cancer information, noting that interpersonal communication might have engendered greater confusion based on the exchange of inaccurate or incomplete information. Recently, Yang et al. [37] also demonstrated that reducing excessive discussion contributes to an individual’s psychological well-being during the COVID-19 pandemic. However, according to Ji et al. [18], people become more selective and efficient in information searching when encountering information overload. Thus, it is also feasible that people seek advice and share information with family and friends, as a trustworthy source, to resolve the confusion and distress caused by the overload of information. Chae et al. [3] also proposed a buffering effect of social support from interpersonal networks by showing that lack of personal resources (represented by cancer information overload and cancer fatalism) led to information avoidance only for those with fewer close friends. Chae et al. [3] stated that social support protects people from the negative emotional states induced by cancer information exposure. Likewise, it might be possible that those suffering from information overload engaged in interpersonal communication more actively in the times of COVID-19 as well. If that is the case, interpersonal communication would be an effect of information overload, rather than a cause, and therefore, needs further in-depth investigation.

As such, one of the major issues in this line of research is the complexity between information overload and information-related behavior (e.g., searching, avoidance) because one can affect the other and vice versa. In other words, frequent media use is likely to increase the amount of information to process, which makes the probability of information overload higher (i.e., a positive association). At the same time, however, overloaded people intentionally avoid information by not using media (i.e., a negative association). It seems that the forces in the two directions may have balanced out in the current study. All of these conjectures deserve deeper investigations by examining the influence of other individual attributes (e.g., information needs, motivation to process information) and information characteristics (e.g., the complexity).

### 5.2. Consequences of Information Overload

Unlike the consistent relationships found in previous studies, there was no direct impact between information overload and intentions to take prevention behaviors in the current study. For example, this finding does not concur with that from Chon and Park’s [38] study showing that engagement in information-related behaviors by the public (e.g., taking and transmitting information about an infectious disease) is positively related to their behavioral intentions to follow the CDC’s instructions during a hypothetical disease outbreak. In the context of COVID-19, Liu [39] found that, among people in Singapore, information seeking via social and online news media was directly associated with their preventive behaviors, and concluded that this may have originated from intense worry elicited by the information.

The non-significant impact on behavioral intention as found in this study seems to result at least partially from the highly skewed distribution and low variance for the behavioral intention variable (*M* = 4.44; *SD* = 0.612), which hinders statistical significance. As mentioned earlier, the survey was conducted in the middle of intensive public and media attention to COVID-19, and a strong third variable or variables about individuals (e.g., risk perception, fear) or the environment (e.g., the intensity of media coverage, the number of cases and mortality, government’s prevention guidance) might have affected the respondents’ strong tendency to engage in prevention behaviors. In this regard, it is important to take the ‘context’ into account in examining the phenomenon of information overload, as Kim et al. [9] emphasized.

The current study found an indirect influence of information overload on behavioral intention via two modes of information processing. Information overload significantly increased heuristic processing while decreasing systematic processing. Furthermore, heuristic processing discouraged behavioral intentions while systematic processing encouraged it. It is worth noting that, through the accumulation of heuristic processing, information overload potentially influences the weakening of one’s willingness to engage in prevention behaviors because judgments based on heuristic processing tend to be “less stable and less tied to subsequent behaviors” ([32], p. 368). Presumably, such an effect will be visible when the panic associated with this global pandemic fades out over time. Thus, future studies are desirable to observe whether the current results are consistent across different contexts and time frames.

Although it was beyond the scope of current study, it is worth noting that scholars (e.g., [40]) and authorities in the international health organizations (e.g., [41]) have expressed great concerns over the phenomenon of infodemic (coined as a blend of information and epidemic) and its negative outcomes, such as the spread of rumors and inaccurate information, decline in news credibility, and confusion over how to behave to prevent the disease. Responding to their calls for public and scholarly attention to be paid to these critical issues [40,41], future studies would be prudent to examine the role of the media in acting against rumors and misinformation, and examine the impact of the media on individuals. In addition, in light of the psychological and emotional impacts of COVID-19 information (e.g., fatigue and anxiety) found in the recent studies [37,42,43], future studies would add more valuable insights if they aim to investigate psychological and emotional responses of information overload.

### 5.3. Limitations of the Study and Suggestions for Future Studies

The current study bears a few limitations that merit future studies. First, while focusing on selected predictors, the influence of other confounders was not explored in this study. For example, as shown in Hsu et al. [44], pre-existing mental and physical health status may play an important role given that contracting COVID-19 directly threatens one’s health condition. In a similar vein, as the workplace is where people spend most of their time, employment status and type of occupation may have a significant association with how an individual perceives the threat of COVID-19 and processes relevant information [44]. It is also possible that parents with a young child may be more sensitive to the threats from COVID-19 and accordingly, be more attentive to relevant information. In addition, considering the substantial influence of cognitive capacity (i.e., current knowledge and information ability) found in this study, information overload may be highly associated with high levels of uncertainty, ambiguity, and the complexity of information regarding COVID-19. In particular, an individual’s familiarity with scientific jargon may affect how easily they process the information, given that information about COVID-19 typically contains a lot of scientific jargon. Although this study employed a measure of ‘information ability’ to assess an individual’s subjective evaluation of their understanding of technical information, it could be more usefully measured in a more objective manner. Future studies should further investigate the role of these variables for their potential influence on the phenomenon regarding information overload.

Second, the sample in this study was limited to residents of Seoul, which is inhabited by younger and more educated individuals. Accordingly, those who were more educated were over-represented in the sample as compared to the entire South Korean population. This seems to largely result from the method of data collection (i.e., the use of online survey) and the greater sample size for the group aged 20 to 49 years than the group of those aged 50 years or over. These sample characteristics suggest caution in the interpretation of the results. The results might have differed if the survey was conducted in another area of South Korea, which has a different socio-demographic composition.

Regarding data collection, this study was conducted in South Korea at the very beginning of the COVID-19 pandemic. This was before COVID-19 severely affected the Western countries of Europe and America. These factors may have created a unique study context, indicating the need for cautious interpretation of the findings and further investigations because they may not be applicable to other contexts. Therefore, it is expected that future studies will be conducted in different contexts, for example, in terms of the phase of the pandemic, nation-wide impacts of COVID-19 and the government’s handling of it.

Lastly, the media use variable deserves further elaboration. Even within a single media outlet, different types of content and information are provided to users and require a varying degree of activeness. For example, even though social media is known as an interactive media platform, people are often exposed incidentally to news in social media [45]. Such patterns of media usage cannot be captured accurately by measuring the mere frequency of using each media channel as in the current study. As information overload is highly associated with the cognitive resources available, the route of information acquisition (e.g., active search vs. passive exposure) should be taken into account in future studies. In addition, the integration of emotional aspects into the investigation will be meaningful. Because news stories about COVID-19 often contain emotionally arousing components that require additional cognitive resources, examining the effect of emotion-laded information on information overload and processing would be an interesting and valuable addition to this line of inquiry.

## 6. Conclusions

In fighting COVID-19 over the past several months, many people have become tired of performing prevention behaviors such as social distancing and wearing a mask, and they are becoming insensitive to the recommendations against COVID-19, claiming fatigue from too much information. However, as an inverted U-shape indicates [18], it would be a difficult task, if not impossible, to pinpoint how just much information is “too much” and to identify what makes people suffer overload. Nevertheless, through conducting a survey in South Korea, we found four significant predictors of information overload (i.e., information ability, current knowledge, frequency of interpersonal communication, and frequency of using online news) as well as the direct impacts on information processing and indirect impacts on behavioral intention via information processing.

Based on the results of the current study, we expect special attention now to be paid to the information overload phenomenon. First, it would be important for health communicators and professionals to examine the current information environment, particularly online news platforms. To reduce information overload and the negative consequences associated with it, they should be guided to present succinct content and not over-repeat information. In addition, as many scholars have already pointed out, public education programs would be pertinent in improving an individual’s ability in handling information in this information-overloaded environment.

## Figures and Tables

**Table 1:** Hierarchical regression results.

Factors	Model 1			Model 2		
	*β*	*t*	*p*	*β*	*t*	*p*
Socio-Demographics						
Gender	0.030	0.736	0.462	0.019	0.541	0.589
Age	−0.025	−0.633	0.527	0.024	0.670	0.503
Level of Education	−0.017	−0.405	0.686	−0.034	−0.971	0.332
Level of Household Income	−0.001	−0.018	0.985	0.014	0.404	0.686
Cognitive Capacity						
Current Knowledge				−0.073	−1.976	0.049
Information Ability				−0.519	−13.753	<0.001
Media Use						
Interpersonal Communication				0.115	3.268	0.001
Television News				0.025	0.658	0.511
Newspaper				0.005	0.141	0.888
Online News Channels				0.100	2.725	0.007
Social Media				0.057	1.577	0.115
Mobile Applications				−0.056	−1.425	0.155
Government Channels				0.049	1.300	0.194
	*F* = 0.301, *p* = 0.878 *R*^2^ = 0.002			*F* = 21.371, *p* < 0.001 *R*^2^ = 0.312 Δ*R*^2^ = 0.310 (*p* < 0.001)		

**Table 2:** Results of structural equation modeling.

			*β*	*B*	*S.E.*	*C.R.*	*p*
Heuristic processing	←	Information overload	0.296	0.416	0.066	6.310	<0.001
Systematic processing	←	Information overload	−0.113	−0.117	0.049	−2.373	0.018
Behavioral intention	←	Information overload	0.063	0.046	0.032	1.445	0.149
Behavioral intention	←	Systematic processing	0.285	0.199	0.041	4.865	<0.001
Behavioral intention	←	Heuristic processing	−0.237	−0.122	0.029	−4.161	<0.001
IO7	←	Information overload	0.618	1.000			
IO6	←	Information overload	0.734	1.216	0.082	14.777	<0.001
IO5	←	Information overload	0.548	0.876	0.074	11.769	<0.001
IO4	←	Information overload	0.487	0.752	0.071	10.650	<0.001
IO3	←	Information overload	0.825	1.509	0.094	15.988	<0.001
IO2	←	Information overload	0.764	1.260	0.083	15.207	<0.001
IO1	←	Information overload	0.805	1.344	0.085	15.741	<0.001
HP1	←	Heuristic processing	0.850	1.000			
HP2	←	Heuristic processing	0.911	1.072	0.046	23.212	<0.001
HP3	←	Heuristic processing	0.587	0.701	0.046	15.315	<0.001
SP1	←	Systematic processing	0.722	1.000			
SP2	←	Systematic processing	0.832	1.087	0.066	16.447	<0.001
SP3	←	Systematic processing	0.695	0.882	0.058	15.160	<0.001
BI1	←	Behavioral intention	0.538	1.000			
BI2	←	Behavioral intention	0.852	1.352	0.098	13.851	<0.001
BI3	←	Behavioral intention	0.916	1.466	0.105	13.983	<0.001
BI4	←	Behavioral intention	0.726	1.322	0.078	16.961	<0.001
Covariances							
e_sysematic_	↔	e_heuristic_	−0.533	−0.253	0.028	−9.088	<0.001
e_BI1_	↔	e_BI4_	0.478	0.169	0.018	9.630	<0.001
